# *Rosmarinus Officinalis* L. essential oil: in Silico molecular docking and in vivo nematocidal activity on intestinal and muscular trichinellosis

**DOI:** 10.1186/s12906-025-04996-7

**Published:** 2025-07-09

**Authors:** Sara A. Abdel Salam, Salama M. El-Darier, Mohamed I. Ashmawy, Marwa Abdelaziz, Fadwa M. Arafa

**Affiliations:** 1 Department of Medical Parasitology, Faculty of Medicine, Alexandria University, Alexandria, Egypt; 2 Department of Botany and Microbiology, Faculty of Science, Alexandria University, Alexandria, Egypt; 3 Department of Pharmaceutical Chemistry, Faculty of Pharmacy, Alexandria University, Alexandria, Egypt; 4 Department of Pathology, Faculty of Medicine, Alexandria University, Alexandria, Egypt

**Keywords:** *Trichinella spiralis*, *Rosmarinus officinalis* essential oil, Molecular Docking, Antioxidant, Anti-inflammatory, Antifibrotic

## Abstract

**Background:**

Despite its deleterious effects, modest activity in muscular larval stages and burgeoning resistance, the control of trichinellosis relies primarily on albendazole. To avert the shortfalls, *Rosmarinus officinalis* essential oil (ROEO), a promising traditional African phytotherapeutic agent, has been harnessed to evaluate its therapeutic profile on murine trichinellosis.

**Methods:**

Employing in silico molecular docking, the interaction between phytochemicals of ROEO and possible targets, tubulin tyrosine ligase and thymidylate synthase was explored. The in vivo anti-*Trichinella* activity of a five-day oral treatment of 400 mg/kg/dose of ROEO was assessed against adult and larval stages via parasitological, ultrastructural, biochemical and histopathological studies.

**Results:**

Molecular docking analysis revealed that the main constituents of ROEO exhibited variable interactions with both targets. Parasitologically, ROEO administration recorded a statistically significant reduction (84.0%R and 73.5%R) in mean worms recovered during the intestinal and muscular phases, respectively. Severe topographic deformities were observed in ROEO-treated worms. Biochemically, the highest antioxidant reduced glutathione level that constrained the detrimental oxidant malondialdehyde in serum was achieved by ROEO. Histopathologically, ROEO had obviously ameliorated inflammatory and fibrotic responses during the muscular stage.

**Conclusions:**

This study is the first to highlight the in silico molecular docking and in vivo anti-*Trichinella* activity of ROEO. Its potentiality in inducing multistage activity and mitigating the inflammation in both intestine and muscles was greatly indebted to suppressing targeted proteins and its high antioxidant activity.

## Background

Trichinellosis, a cosmopolitan anthropozoonosis, is most often caused by *Trichinella spiralis* (*T. spiralis*) species belonging to the polytypic genus of *Trichinella* [1]. Humans become infected after consumption of improperly cooked meat of domestic or game ungulates containing encysted first stage *Trichinella* larvae [2]. Although *T. spiralis* infection has been documented in different mammalian hosts, only humans are likely to develop an overt clinical disease [3]. The clinical course of the disease typically progresses from enteral into parental stages [4]. Although the majority of these cases were asymptomatic, life-threatening complications as myocarditis, encephalitis, pneumonia, renal failure, vascular thrombosis or hypokalaemia may occasionally develop [1, 5].

Albendazole (ALB) and mebendazole, members of benzimidazole family, are the principal medications for trichinellosis treatment [4]. Evidently, ALB is much more efficient in combating the intestinal adult worms than both newborn and encysted muscle larvae [5]. The serious adverse effect profile, poor water solubility and bioavailability as well as the prevailing emerging resistance of ALB ultimately limited its therapeutic efficiency [6, 7]. Despite implemented measures to control *T. spiralis* infection, the complexity of its life cycle remains a major stumbling block to the development of an ideal vaccine [8]. In a quest to curtail the long-voiced concerns associated with ALB, the discovery of alternative natural efficient therapeutics against trichinellosis is imperative and indispensable in the prime time.

Since antiquity, the public health and environmental benefits of traditional alternative medicine, particularly phytomedicine and its derivatives have been witnessed [9, 10]. The global paradigm shift from the trendy synthetic medications to herbal medicine may imply that the latter has minimal adverse impacts with better compliance, good biocompatibility, and lesser cost [11]. A plethora of medicinal herbal extracts, bioactive compounds and essential oils, have been exploited in human health improvement and treatment of various diseases [12, 13].

*Rosmarinus officinalis* (RO) L. species of genus *Rosmarinus*, family Lamiaceae (Labiatae), is one of the most popular perennial evergreen woody native Mediterranean aromatic under-shrub [14]. Since ancient times, the traditional African RO has been widely used in culinary, cosmetic and medicinal purposes owing to its high nutritional value and ethnopharmacological attributes [15]. Apart from its nutritional values, ROEO has been exploited in the treatment of hypertension, diabetes, cancers, respiratory and digestive disorders, kidney stones, dysmenorrhea and wound healing [14]. Consistent with the high antioxidant capacity of ROEO, its antimicrobial activity against different pathogenic bacteria [16, 17], fungi [18, 19] and viruses were documented [20]. In terms of parasitic diseases, it exhibited in vitro amoebicidal [21], trypanocidal, leishmanicidal [22], trichomonicidal [19] as well as coccicidal activities [23]. In addition, ROEO was potentially fruitful in preclinical in vivo models as it held promising antiparasitic activity against *Trypanosoma evansi* [24, 25] and cystogenic ME49 strain of *Toxoplasma gondii* [26].

Accumulated evidence suggested its promising nematocidal effect in vitro. For instance, recent studies have highlighted its significant in vitro ovicidal and larvicidal properties, particularly against gastrointestinal nematode of sheep, *Haemonchus contortus*, inhibiting the egg hatching and motility of adult worms, demonstrating strong anthelmintic properties [23, 27]. Moreover, ROEO has served as an effective, natural and environmentally friendly nematocidal agent in agriculture due to its notable effects against plant nematodes [28]. The scarcity of in vivo studies in this field encouraged the authors to pursue with the current research to evaluate its activity against the parasitic nematode, *T. spiralis* in mice.

As such, considering the witnessed activity of ROEO as a natural phytotherapeutic agent in curbing a vast array of parasitic diseases, its therapeutic efficacy was rigorously evaluated against intestinal and muscular trichinellosis in swiss mice model via parasitological, ultrastructural, histopathological and biochemical studies. Apart from the in vivo study, exploring the possible mechanism of action through an in silico molecular docking study was performed.

## Methods

### Preparation and extraction of ROEO

The leaves of RO were collected from a private farm in Borg El-Arab, Alexandria, Egypt in February 2023 after obtaining an oral permission from the landowner. The plant was identified by Salama M. El Darier, Professor of Plant Ecology, Department of Botany and Microbiology, Faculty of Science, Alexandria University and deposited at the Herbarium of Alexandria University (Voucher No. 4114). The leaves were selected, rinsed, and left to dry at room temperature for five days before grinding. By moving steam generated in one round-bottomed flask into another 3-liter flask holding 1 kg of pure ground rosemary leaves and 1 L of distilled water, the essential oil was extracted via hydro-distillation. The steam travelled through the plant and was charged with essential oil to form a water-oil vapor mixture before condensation. To extract the maximum oil possible, the procedure was done for two hours, and repeated until no more oil remains. Following condensation, the water-oil condensate was gathered in a flask with a round bottom. After each fraction, the oil and water were separated by decantation. The essential oil was finally dried on anhydrous sodium sulfate and kept at 4 °C [9].

### In silico molecular docking study

Building upon gas chromatograph-mass spectrometer analysis of ROEO, extracted from leaves of RO brought from same farm in a prior work [9], the most prominent constituents from ROEO were selected for molecular docking experiments to investigate the potential target and binding site within protein scaffolds and subsequently assess ROEO’s effectiveness against *T. spiralis* as a therapeutic agent. Two possible targets are tubulin tyrosine ligase [29] and thymidylate synthase [30] were identified based on the biological and in silico studies. Docking studies were carried out using MOE 2020.09 software. The compounds were retrieved from PubChem and prepared by adding hydrogens, calculating partial charges, and minimizing energy with Amber10: EHT Force Field and a root mean square (RMSD) gradient of 0.1 kcal/mol. Furthermore, the preparation of proteins involved eliminating repetitive chains and water molecules. The MOE QuickPrep procedure optimized structural issues, performed 3D protonation, and calculated partial charges. The MOE Dock technique was utilized to find the optimal postures and binding score parameters for the selected compounds through induced fit refinement. In order to validate our docking strategy, the co-crystallized ligands TPI-1 [31] for tubulin tyrosine ligase (PDB ID: 5XLZ) and deoxyuridine monophosphate (dUMP) for thymidylate synthase (PDB ID: 5BY6) were re-docked into their active site, respectively and RMSD values were less than 2. The triangle matcher placement technique and London dG served as the compound’s principal scoring functions. An additional refinement step was performed using the induced fit technique and the affinity dG score function. Furthermore, docking positions were assessed, and interactions with the active site were studied. Finally, the postures with the highest score that plugged into the active site and had advantageous ligand-enzyme interactions were selected.

### In vivo animal experimentation

#### Parasite

The strain of *T. spiralis* used in the current study (Istituto Superiore di Sanita code: ISS6158) was initially provided by the parasitology division of Theodor Bilharz Research Institute in Giza, Egypt. Then, the strain was maintained in the Medical Parasitology Department, Faculty of Medicine, Alexandria University, Egypt by serial passage in Swiss strain albino mice.

#### Animal grouping and experimental design

The present work was conducted on 42 Swiss albino mice, aged between 6 and 8 weeks with a weight in the range of 20–25 g, obtained from the animal house of the Medical Parasitology Department, Faculty of Medicine, Alexandria University. Animals were fed a standard pellet diet and water ad libitum. They were housed in a clean polypropylene cage with a perforated cover in a well-ventilated room (25 ± 2 ^o^C) and maintained on 12:12 h light: dark cycle at the animal house. All mice were subdivided into four main groups. Group I: 6 non-infected, non-treated mice; Group II: 12 infected, non-treated mice; Group III: 12 infected ALB-treated mice (Alzentale^®^ given orally at 50 mg/kg/dose as a suspension in distilled water) [32]; Group IV: 12 infected ROEO-treated mice (given orally at 400 mg/kg/dose emulsified in 0.5% Tween-80 solution) [26].

In infected groups (II, III and IV), each mouse was orally infected with 250 larvae via gastric gavage [33]. Mice in these infected groups were equally subdivided. Half of the mice in groups III and IV initiated the five-day treatment regimen on the same day of infection, while the remaining ones started the treatment on the 23rd day postinfection (dpi). The appearance of any of the following was considered a humane endpoint: a reduction of over 20% of the initial body weight; an inability to ambulate; signs of respiratory distress; signs of sickness behaviour (lethargy, sleepiness, anorexia or inability to respond to gentle prodding) [34]. Mice in all infected groups (II, III and IV) were sacrificed at two different evaluation times: on the 5th dpi to assess the effect on the intestinal phase and on the 28th dpi to determine the effect on the muscular phase. They were anaesthetised by intraperitoneal injection of 40 mg/kg of sodium pentobarbital before blood collection from the jugular veins. Afterwards, the unconscious mice were sacrificed by cervical dislocation. Death was confirmed after euthanasia by cessation of heartbeats and respiration, toe pinch reflexes loss, mucous membranes greying, or rigor mortis.

### Assessment of the effect of treatment

#### Parasitological study

In infected groups (II, III and IV), adult worm count was performed after two-hour incubation of the small intestine of one-half of mice on the 5th dpi, while larvae were counted in the remaining mice on the 28th dpi after two-hour of chlorhydropeptic artificial digestion [33].

The reduction in parasite burden was determined according to the following equation:

$${\text{Percentage}}\:{\text{reduction}}\left( {\% {\text{R}}} \right) = \frac{{{\text{N}} - {\text{n}}}}{{\text{N}}} \times 100$$

*N*: Mean adult/larval count recovered from the infected non-treated group (II).

*n*: Mean adult/larval count recovered infected treated group.

#### Ultrastructural study

Adult worms and larvae collected from the small intestine and diaphragm of mice in the infected groups (II, III and IV) were fixed in cold 2.5% buffered glutaraldehyde phosphate, dehydrated, and examined under scanning electron microscopy (SEM) (JEOL JSM, IT200, Japan) [13].

#### Histopathological study

As regards the intestinal changes caused by the adult worms on the 5th dpi, which corresponds to the peak of the histopathological alterations caused by the infection, one-centimeter sections of the jejunum from each mouse of the infected groups (II, III, and IV) were excised following sacrifice and preserved in 10% formalin. After being longitudinally cut, the segments were placed on their sides in plastic cassettes and processed to paraffin blocks. Five-micron thick sections were then cut with at least three serial sections prepared from each block, mounted on glass slides and finally stained with Hematoxylin and eosin (H&E). Then, the slides were assessed blindly by an expert evaluator using light microscopy. To study the alterations in the jejunum, a total of ten low-power fields (LPF) per mouse were examined to assess damage, inflammatory cellular infiltrate and density of goblet cells. A scoring system, that ranged from 0 to 4, was used to assess jejunal tissue damage where 0 represented normal tissue pattern with normal villous to crypt ratio; 1 represented minimal shortening and broadening of villi and crypt hyperplasia; 2 represented mild shortening and broadening of villi and crypt hyperplasia; 3 represented obvious shortening and broadening of villi and crypt hyperplasia; and 4 represented extensive mucosal damage extending through deeper structures of the intestinal wall. Additionally, another scoring system ranging from 0 to 4 was used to assess the infiltration of inflammatory cells in the jejunum where 0 represented normal cell type pattern; 1 represented scattered inflammatory cells in the lamina propria; 2 represented numerous inflammatory cells in the lamina propria; 3 represented the confluence of inflammatory cells extending into the submucosa; and 4 represented transmural extension of the inflammatory cells [35]. The mean number of jejunal goblet cells per 10–15 enterocytes was calculated. Then, according to this mean number, they were divided into three categories: goblet cell depletion (mean goblet cells < 1/10–15 enterocytes), average goblet cells (mean goblet cells = 1/10–15 enterocytes) and goblet cell hyperplasia (mean goblet cells > 1/10–15 enterocytes) [33].

On the other hand, diaphragmatic sections were stained with H&E and Masson trichrome and examined for determining larval density, inflammatory cellular infiltrate and fibrosis. Ten LPFs were used to estimate the density of muscle larvae and score larval numbers as follows; 1 = < 5 larvae/ LPF, 2 = 5–10 larvae/field, and 3 = > 10 larvae/field [36]. The density of the inflammatory cellular infiltrate around the larval capsules was rated in the following manner: 1 denoted a mild infiltrate, 2 a moderate infiltrate, and 3 an intense infiltrate [36]. Masson trichrome-stained muscle sections were used to evaluate the integrity and thickness of the larval capsule [37].

#### Biochemical study

Malondialdehyde (MDA) and reduced glutathione (GSH) serum levels were determined colorimetrically in the sera of mice in all studied groups using commercial kits (CAT. No. MD2529 and GR2511, Biodiagnostic, Egypt) according to the instructions of the manufacturer.

### Statistical analysis

The experimental data were analyzed via IBM SPSS software package version 20.0. (Armonk, NY: IBM Corp). The normality of quantitative data was tested using the Shapiro-Wilk test and expressed as mean, standard deviation, median, minimum and maximum. For comparison between the studied groups, One way ANOVA test was utilized, while Post Hoc test (Tukey) was performed for pairwise comparison. The statistical level of significance was set at the 5% level.

## Results

### In silico molecular docking study

**Fig. 1:**
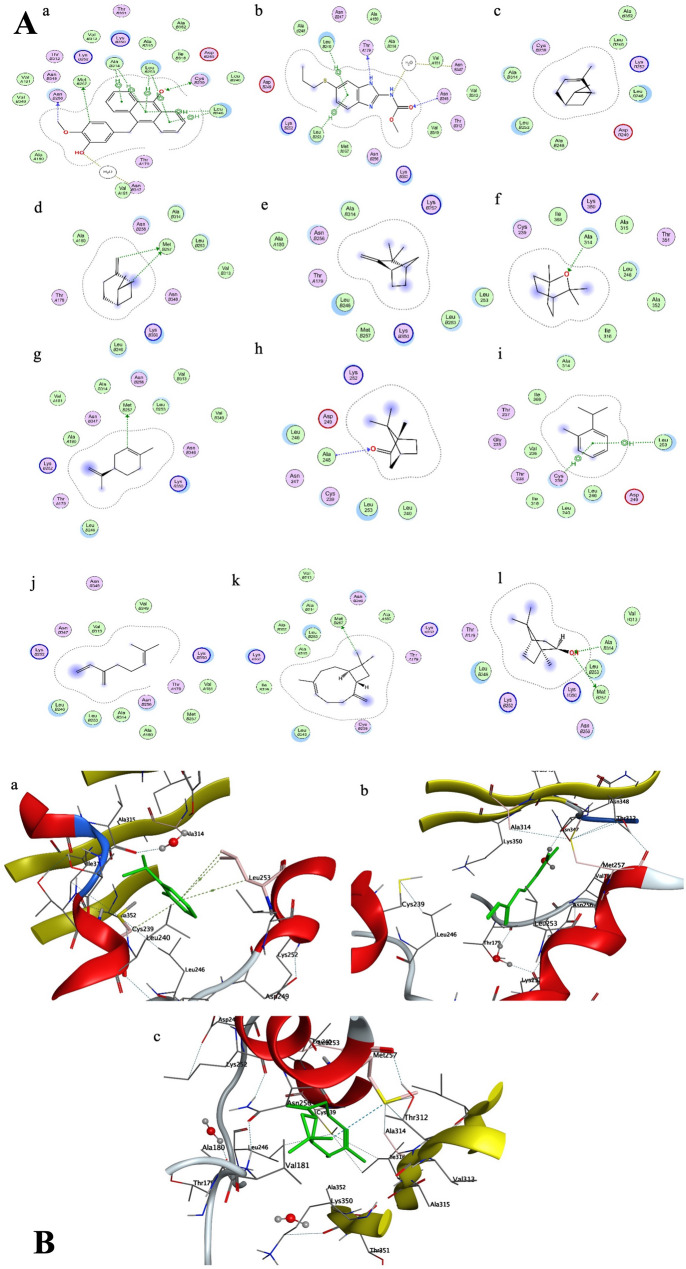
(**A**) 2D ligand-protein interactions of (**a**) TPI-1, (**b**) ALB, (**c**) α-Pinene, (**d**) β-Pinene, (**e**) Camphene, (**f**) 1,8-cineole, (**g**) D-Limonene, (**h**) Camphor, (**i**) O-cymene, (**j**) β-Myrcene, (**k**) Caryophyllene, (**l**) Isoborneol with 5XLZ. (**B**) 3D poses of the most favorable and lower binding energy compounds for tubulin tyrosine ligase: (**a**) O-Cymene, (**b**) β-Myrcene, (**c**) Caryophyllene

The ROEO was compared to ALB, an efficient anthelmintic medicine that targets the parasite’s β-tubulin and inhibits microtubule polymerization; thus, we used PDB ID 5XLZ as a potential target. To evaluate the binding affinity in comparison with ALB, the primary components of essential oil as well as ALB were docked into the tubulin binding site of colchicine [38]. Docking results demonstrated that all major ROEO constituents occupied the same binding pocket as ALB, with slightly higher binding energy with ALB (-7.76 Kcal/mol) than ROEO (ranging from − 6.65 to -4.82 Kcal/mol). The co-crystallized ligand’s (TPI-1) primary interactions were hydrogen bonds with Thr179, Asn347 (via a water bridge), Met257, and Cys239, as well as hydrophobic interactions with Leu246, Leu253, and Ala314 as depicted in Fig. 1A. ALB showed interaction through hydrogen bonds with Thr179, Asn348, and Asn347 (via a water bridge) as well as lipophilic interactions with Leu246 and Leu253 as shown in Table 1; Fig. 1A & B. The constituents of ROEO exhibited a variety of interactions, including hydrophobic interactions with Cys239 and Leu253, and hydrogen bonding interactions with Ala248, Leu253, and Met257. Caryophyllene, β-Myrcene, and O-Cymene showed the lowest and the most favorable binding score to the protein (S-score = -6.65, -5.82, and − 5.73 Kcal/mol, respectively).

**Table 1:** Binding scores and amino acid of enzymes involved in interactions with main components of ROEO*

ligand	5XLZ		5BY6	
	Binding scores ΔG (Kcal/mol)	Amino acid interaction and distance in (A˚)	Binding scores ΔG (Kcal/mol)	Amino acid interaction and distance in A˚
α-Pinene	-5.53	Ligand exposure	-4.63	Cys189 (3.92) His250 (3.72)
β-Pinene	-5.63	Met257 (4.5)	-4.62	Trp103 (3.59)
Camphene	-5.45	Ligand exposure	-4.53	Ligand exposure
1,8-Cineole	-5.11	Ala314 (2.70)	-4.59	Cys189 (3.68)
D-Limonene	-5.65	Met257 (3.85)	-4.52	His250 (4.01)
Camphor	-4.96	Ala248 (3.62)	-4.33	Phe219 (3.86) Asn220 (3.38)
O-Cymene	-5.73	Cys239 (3.57) Leu253 (3.89)	-4.35	Arg43 (3.95) Arg209 (4.68) His250 (2.80)
β-Myrcene	-5.82	Ligand exposure	-4.66	Cys189 (3.77) Phe219 (4.61)
Caryophyllene	-6.65	Met257 (3.82)	-5.22	Trp103 (3.56)
Isoborneol	-4.82	Met257 (4.15) Ala314 (2.90)	-4.26	His250 (2.80) Tyr252 (2.78)
TPI-1	-8.62	Cys239 (3.06) Met257 (3.82) HOH630 (2.78) Asn256 (3.52) Leu246 (3.86) Leu253 (3.97) Ala314 (3.65)	-----	--------------
ALB*	-7.76	Thr179 (3.38) Leu246 (4.02) Leu253 (4.62) Asn348 (3.29) HOH630 (3.02)	-----	---------------
dUMP	------	-----------	-8.56	Arg43 (2.76) Arg209 (2.69) Ser210 (2.57) Asp212 (2.81) Asn220 (2.94) His250 (2.71) Tyr252 (2.70) HOH559 (2.61) HOH640 (2.59) HOH616 (2.75) HOH693 (2.74)
E63	------	---------------	-6.88	Arg43 (4.34) Ala211 (3.51) Asp212 (3.12) Gly216 (3.58) Asn220 (3.30) Asp248 (3.32) His250 (3.20) Tyr252 (4.41) HOH622 (3.23) HOH559 (3.20)

*ROEO: *Rosmarinis officinalis* essential oil

*ALB: Albendazole

**Fig. 2:**
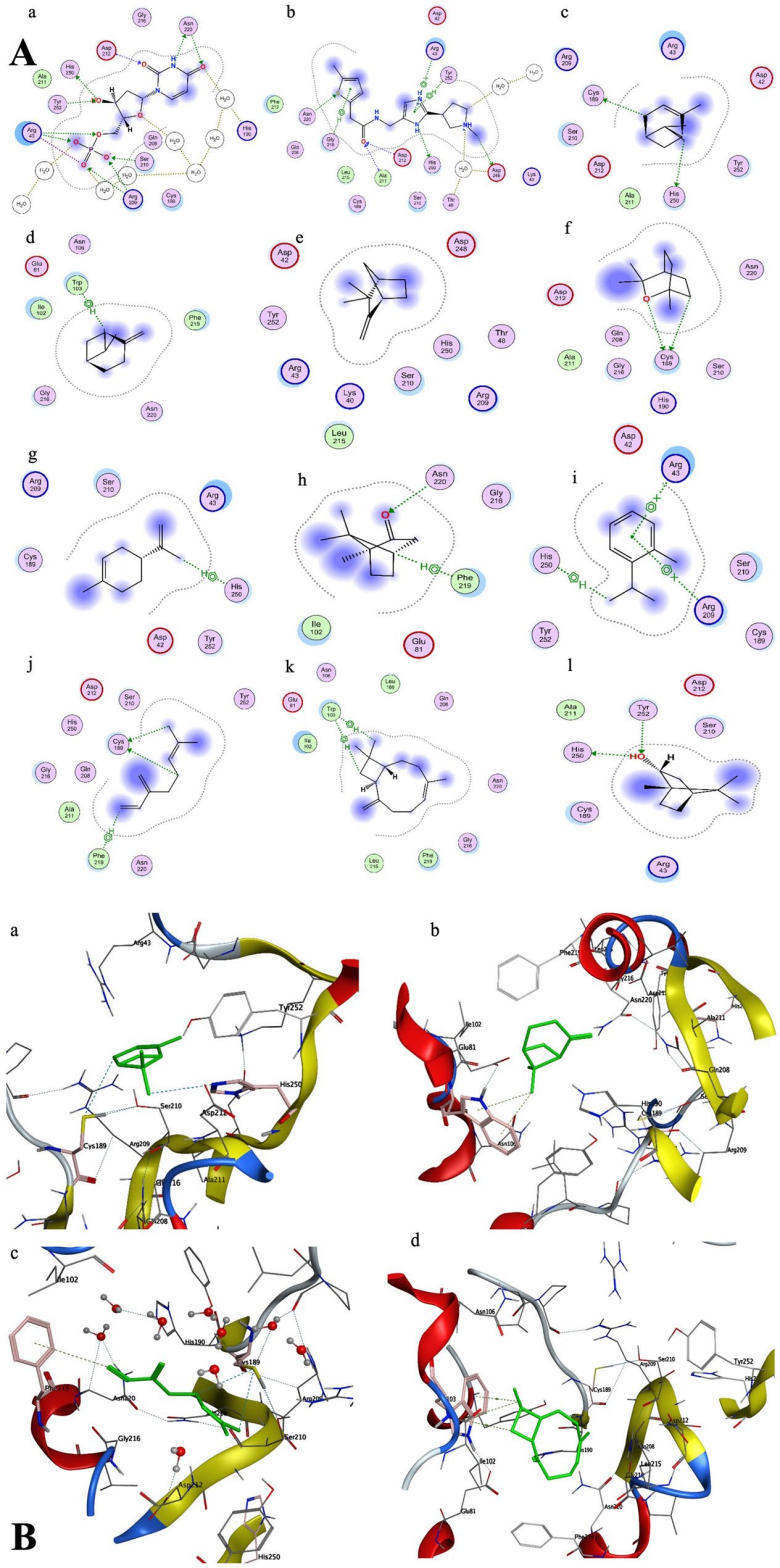
(**A**) 2D ligand-protein interactions of (**a**) dUMP, (**b**) E63, (**c**) α-Pinene, (**d**) β-Pinene, (**e**) Camphene, (**f**) 1,8-cineole, (**g**) D-Limonene, (**h**) Camphor, (**i**) O-cymene, (**j**) β-Myrcene, (**k**) Caryophyllene, (**l**) Isoborneol with 5BY6. (**B**) 3D poses of the most favorable and lower binding energy compounds for thymidylate synthase: (**a**) α-Pinene, (**b**) β-Pinene, (**c**) β-Myrcene, (**d**) Caryophyllene

Inhibiting thymidylate synthase was another potential target found in *T. spiralis* PDB ID: 5BY6. Various inhibitors of thymidylate synthase have been identified including E63 which showed higher selectivity for *T. spiralis* thymidylate synthase. Docking analysis assessing the binding affinity and energy score of E63 to the components of ROEO revealed that E63 has a slightly higher binding affinity and lower energy scores (-6.88 Kcal/mol) than ROEO components (ranging from − 5.22 to -4.26 Kcal/mol). E63 bonded with the thymidylate synthase binding site primarily by hydrogen bonding with Ala211, Asp212, Asn220, Asp248, His250, and Thr48 (via water bridge) and a hydrophobic contact with Gly216, Tyr252, and Arg43 (via pi-cation interaction), while the primary components of ROEO engage with the binding site by means of hydrogen bonding with Cys189, Asn220, His250 and Tyr252. Additionally, they connect with Arg43 and Arg209 by pi-cation and pi-hydrogen bonding with Trp103. Caryophyllene, β-Myrcene, α-Pinene, and β-Pinene exhibited promising binding affinities and energy scores as depicted in Table 1; Fig. 2A & B.

### Parasitological study

Adult worms were collected from intestine of infected mice in groups (II, III and IV) and counted following sacrifice on the 5th dpi. Compared to infected non-treated mice (Group II), both ALB and ROEO achieved a statistically significant reduction in mean adult worm burden 92%R and 84%R, respectively (*P* < 0.001). In the same manner, the mean larval count on the 28th dpi was statistically significantly reduced in both groups (III and IV) compared to the infected non-treated control (Group II) with an 81.7%R and a 73.5%R for ALB and ROEO, respectively (*P* < 0.001). Herein, ALB kept a slightly higher mean larval count than ROEO, yet the difference remained statistically non-significant (Table 2).

**Table 2:** The effect of ALB and ROEO on adult and larval count in different infected groups

Worm count\Infected group	Non-treated (II)	ALB-treated (III)	ROEO-treated (III)	F (*p*)
**Adult**				
Mean ± SD	101.8 ± 5.85	8.17 ± 3.31	16.3 ± 2.34	957.533* (< 0.001*)
Median (Min– Max)	102.5 (93–110)	7.50 (4–13)	17 (13–19)	
% R		**92.0%**	**84.0%**	
Significance	p_1_ < 0.001^*^, p_2_ < 0.001^*^, p_3_ = 0.010^*^			
**Larva**				
Mean ± SD	3628.2 ± 341.4	664.3 ± 90.94	962.5 ± 113.9	347.826* (< 0.001*)
Median (Min– Max)	3572 (3109–4039)	675 (520–781)	957.5 (827–1150)	
% R		**81.7%**	**73.5**	
Significance	p_1_ < 0.001^*^, p_2_ < 0.001^*^, p_3_ = 0.071			

%R: Percentage of reduction in each infected treated group compared to infected non-treated control group (II)

F: for One way ANOVA test, Post Hoc Test (Tukey) for pairwise comparison

p: for comparing between the different studied infected groups

p_1_: for comparing II and III

p_2_: for comparing II and IV

p_3_: for comparing III and IV

*: Statistically significant at *p* ≤ 0.05

### Ultrastructural study

**Fig. 3:**
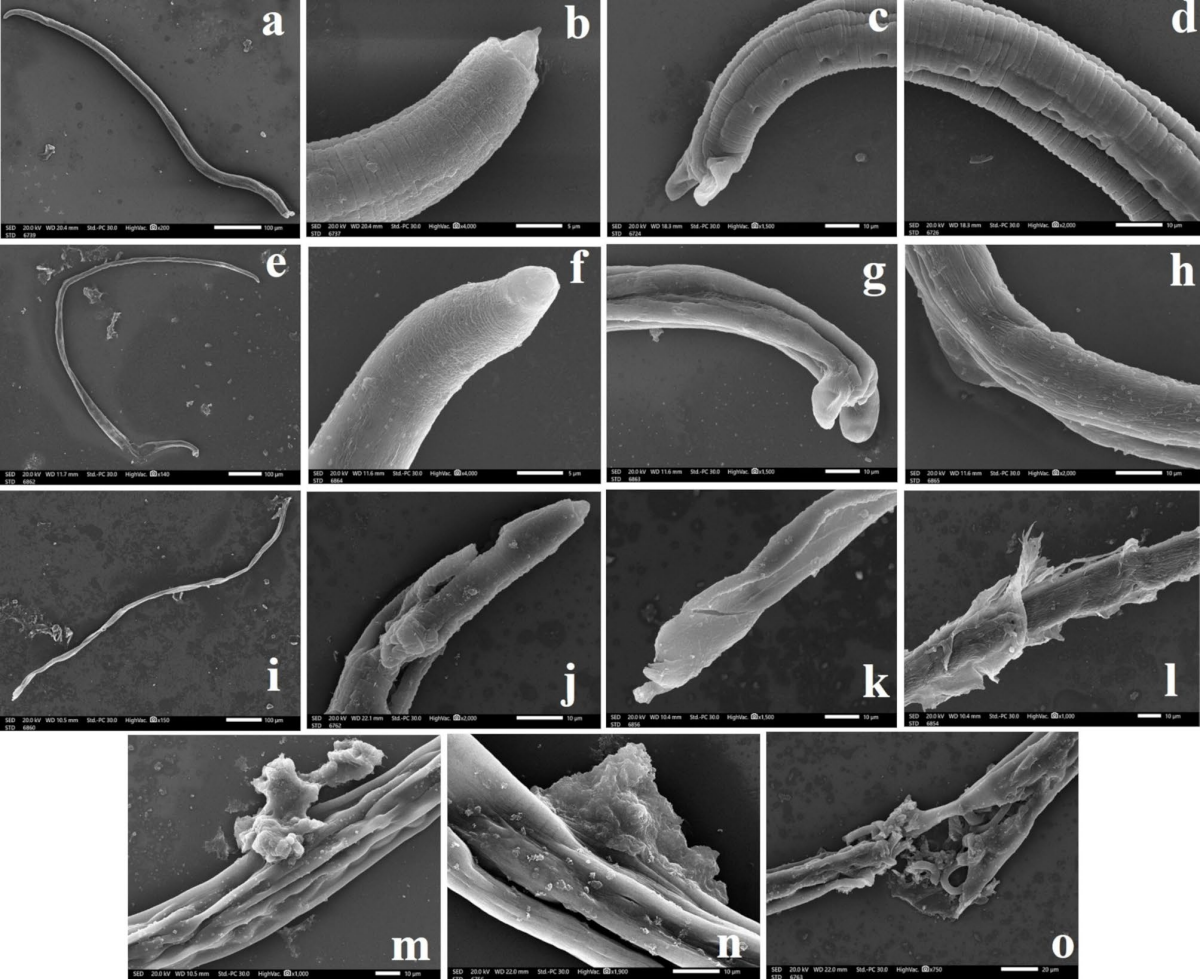
SEM of *T. spiralis* adult worms retrieved from small intestine of infected mice: (**a-d**) non-treated (Group II); (**a**) Cylindrical shaped adult male worm with intact cuticular surface (x200); (**b**) Tapering anterior end with a protruding stylet from the oral opening and evident transverse creases on the tegument (x4000); (**c**) Posterior end showing intact copulatory appendages and a pair of accessory papillae (x1500); (**d**) Mid-section tegumental surface appearing annulated with prominent longitudinal ridges, transverse creases lined up in neat rows, and hypodermal glands’ openings (x2000). (e-h) ALB-treated (Group III); (**e**) Stretched elongated adult male showing partial destruction and incomplete separation of its posterior end (x140); (**f**) Anterior end destroyed with shallow transverse creases (x4000); (**g**) Posterior end with preserved copulatory appendages, yet multiple deepened longitudinal ridges and absent transverse creases (x1500); (**h**) Mid-section tegumental surface showing small tegumental blebs with absent transverse creases (x2000). (i-o) ROEO-treated (Group IV); (**i**) Adult male appeared slender elongated and twisted with compressed body (x150); (**j**) Anterior end with ruptured and discontinuous tegumental surface alongside crater-like depressions (x2000); (**k**) Swollen posterior end with deformed and deflated copulatory appendages (x1500); (**l**) Numerous signs of severely damaged cuticle with sloughed and cast-off cuticle (x1500); (**m**) erupted fungating masses and longitudinal furrows (x1000); (**n**) deep clefts (x1900); (**o**) completely degenerated and ruptured cuticle with exposed internal structures (x750)

On the 5th dpi, SEM examination of *T. spiralis* adult worms retrieved from small intestine of infected, non-treated mice (Group II) showed cylindrical-shaped *T. spiralis* adult male worms with intact cuticular surface, tapering anterior end with occasionally protruding stylets from the oral opening and the posterior end showed copulatory appendages and accessory papillae (Fig. 3a-c). Additionally, the mid-section cuticular surface appeared annulated with prominent longitudinal ridges, transverse creases lined up in neat rows, and hypodermal glands’ openings on the lateral side (Fig. 3d). In contrast, ALB-treated adults (Group III) showed a stretched, elongated adult male with partial destruction and incomplete separation of its posterior end (Fig. 3e). The anterior end of adult males was destroyed (Fig. 3f), while the posterior end had preserved copulatory appendages, yet multiple deepened longitudinal ridges and absent or shallow transverse creases (Fig. 3g). Besides, the mid-section cuticular surface showed small blebs with absent transverse creases (Fig. 3h). Obviously, ROEO-treated adults (Group IV) appeared slender, elongated, and twisted with compressed bodies (Fig. 3i). The anterior end had ruptured and discontinuous cuticular surface alongside crater-like depressions (Fig. 3j), while the posterior end of treated adult males was swollen, with deformed and deflated copulatory appendages (Fig. 3k). Moreover, numerous signs of severely damaged cuticles were detected such as erupted fungating masses, longitudinal furrows, deep clefts, and completely sloughed, cast-off and ruptured cuticle with exposed internal structures (Fig. 3l-o).

**Fig. 4:**
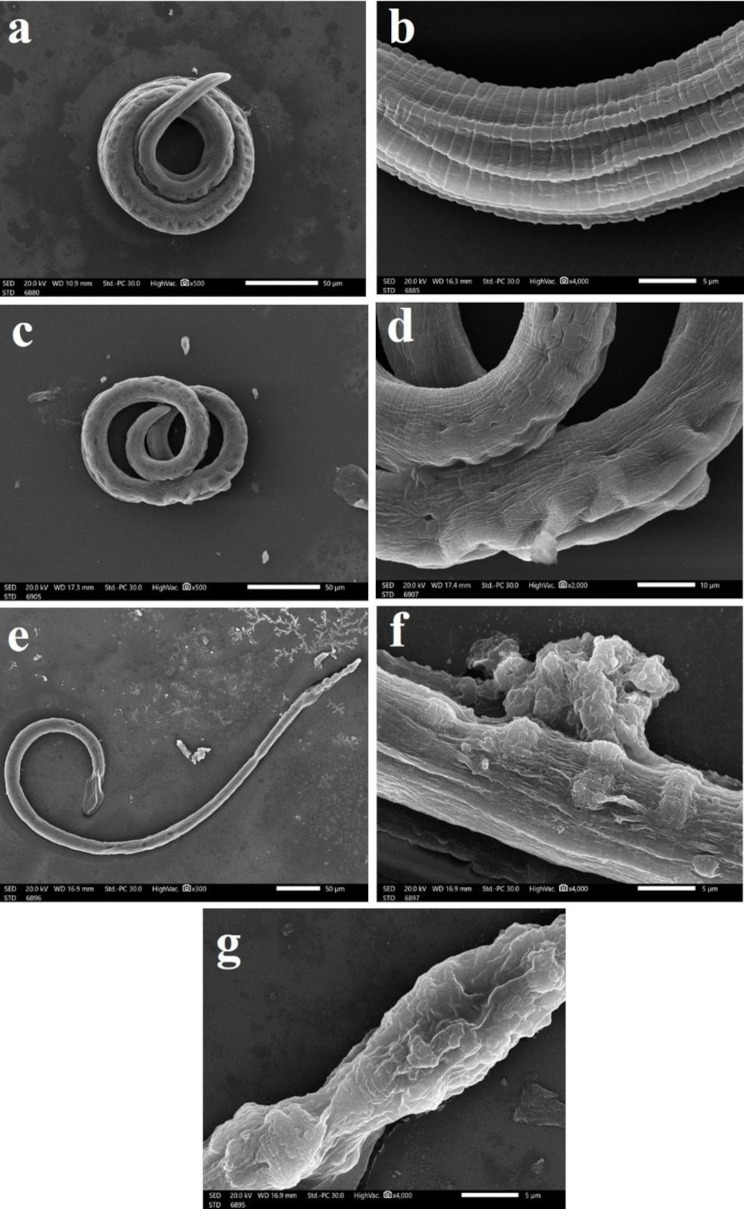
SEM of *T. spiralis* larvae recovered from infected mice: (**a-b**) non-treated (Group II); (**a**) Normal appearance of larva with intact surface, typical coiled manner, and hypodermal glands openings (x500); (**b**) annulated cuticle with shallow parallel longitudinal grooves along the body axis (x4000). (**c-d**) ALB-treated (Group III); (**c**) Larva retained the coiled body with corrugated cuticle (x500); (**d**) Wrinkled larval cuticle with reduced longitudinal ridges and transverse creases (x2000). (**e-g**) ROEO-treated (Group IV); (**e**) Completely flaccid and stretched larva with loss of muscle tone, beaded anterior end, and squashed destructed posterior end (x300); (**f**) Rough tegumental surface, lost annulations, cauliflower-like mass fungating from disrupted rough surface (x4000); (**g**) Destroyed and crumpled cuticle with bodily constriction (x4000)

On the 28th dpi, the micrographs of *T. spiralis* larvae recovered from infected, non-treated mice (Group II) showed normally appearing larvae with intact surfaces, typically coiled manner, hypodermal glands openings and annulated cuticle with shallow parallel longitudinal grooves along the body axis (Fig. 4a and b). Although those collected from ALB-treated larvae (Group III) retained the coiled body, the larval cuticle appeared wrinkled with reduced longitudinal ridges and transverse creases (Fig. 4c and d). While ROEO-treated larvae (Group IVb) looked completely flaccid and stretched larva with loss of muscle tone, beaded anterior end, and squashed destructed posterior end (Fig. 4e). The larvae showed rough cuticular surface with lost annulations, cauliflower-like mass fungating from disrupted rough surface and destroyed crumpled cuticle with bodily constriction (Fig. 4f and g).

### Histopathological study

**Fig. 5:**
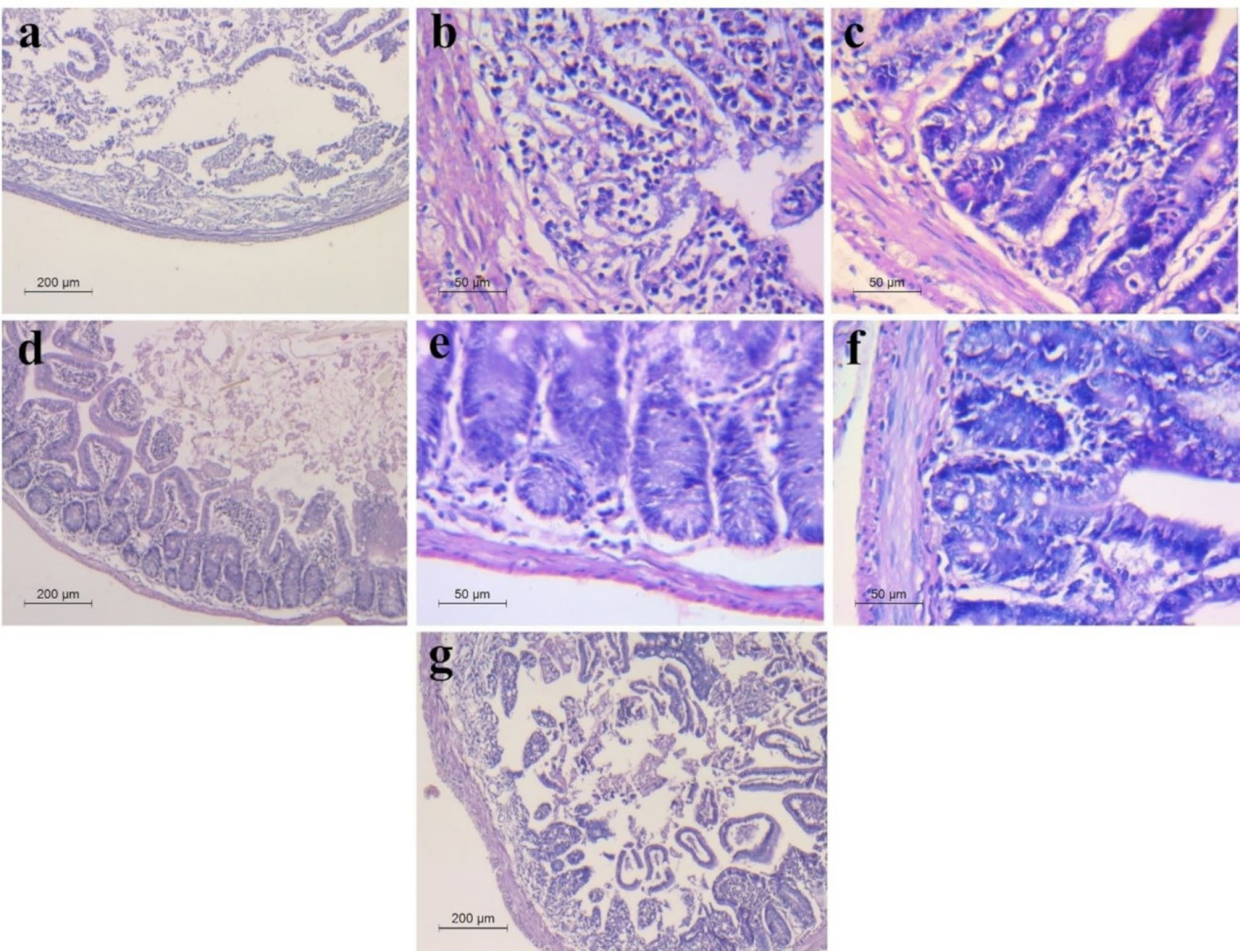
Histopathological findings of H&E-stained jejunal sections of infected mice: (**a-c**) Non-treated (Group II); (**a**) Intestinal section showing mucosal sloughing, evident shortening and broadening of villi and crypt hyperplasia (score 3) (x100); (**b**) Dense inflammatory cellular infiltration extending into the submucosa (score 3) (x400); (**c**) Evident goblet cell hyperplasia (x400). (**d-f**) ALB-treated (Group III); (**d**) Jejunal section showing minimal shortening and broadening of villi and crypt hyperplasia (score 1) (x100); (**e**) scattered inflammatory cells (score 1) (x400); (**f**) Average number of goblet cells (x400). (**g-h**) ROEO-treated (Group IV); (**g**) Jejunal section showing mild shortening and broadening of villi and crypt hyperplasia (score 2) (x100)

Histopathological assessment of H&E-stained jejunal sections of infected, non-treated mice (Group II) showed evident shortening and broadening of villi and crypt hyperplasia (score 3) (Fig. 5a) together with a dense inflammatory cellular infiltration extending into the submucosa (score 3) (Fig. 5b) and evident goblet cell hyperplasia (Fig. 5c). While intestine of infected ALB-treated mice (Group III) displayed minimal shortening and broadening of villi and crypt hyperplasia (score 1) (Fig. 5d) along with only scattered inflammatory cells (score 1) (Fig. 5e) and average number of goblet cells (Fig. 5f). In addition, jejunum of infected ROEO-treated mice (Group IV) revealed mild shortening and broadening of villi and crypt hyperplasia (score 2) (Fig. 5g) with only scattered inflammatory cells (score 1) and restoration of average goblet cell number.

**Fig. 6:**
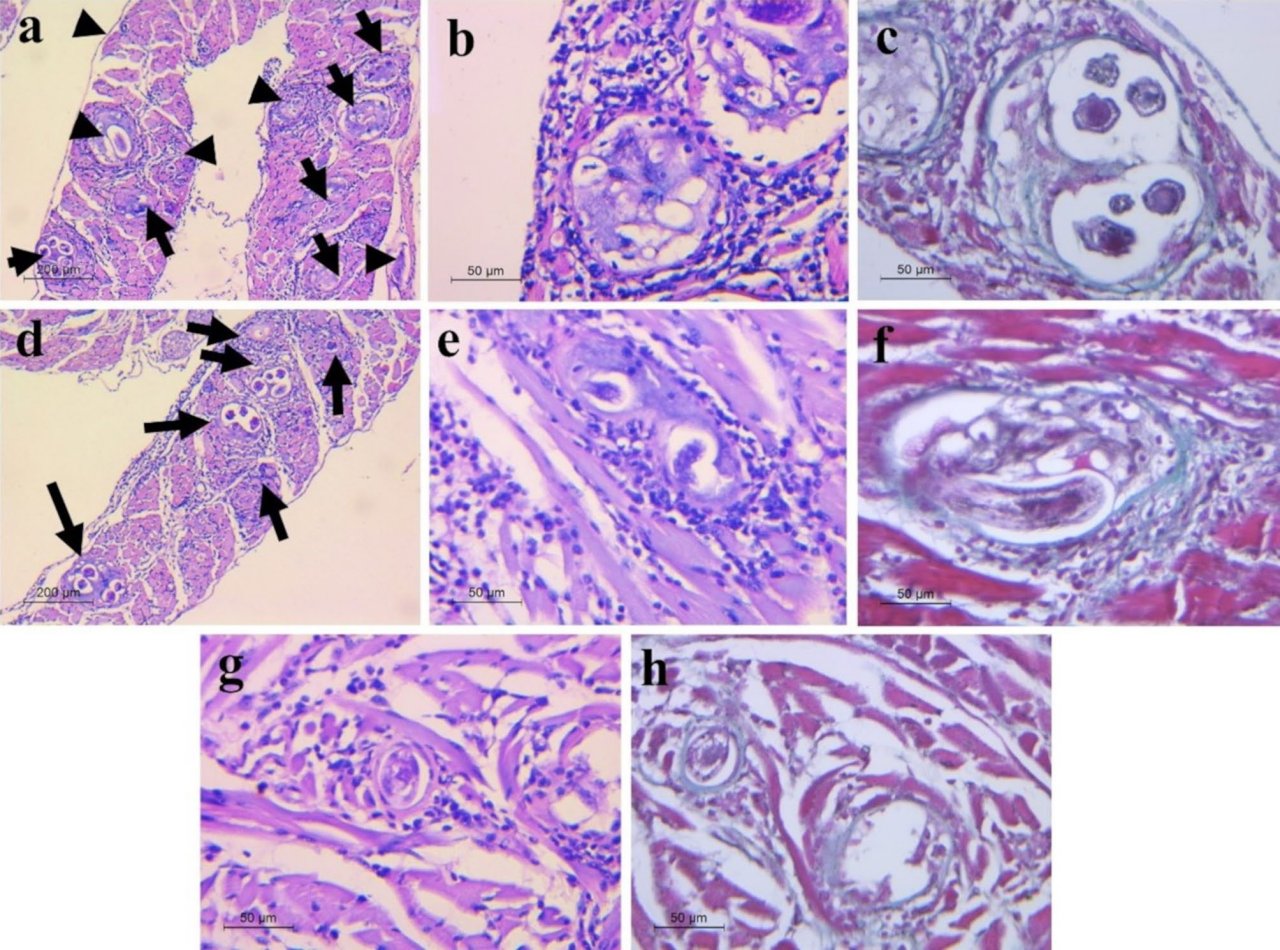
Histopathological findings of diaphragmatic muscle sections of infected mice: (**a-c**) Non-treated (Group II); (**a**) muscle section showing a heavy larval density (black arrows) harboring more than 10 larvae per LPF (score 3) (H&E, x100); (**b**) larvae were surrounded by an intense inflammatory reaction (score 3) (H&E, x400); (**c**) Intact thick well developed larval capsule (Masson trichrome, x400). (**d-f**) ALB-treated (Group III); (**d**) Diaphragmatic section showing fewer larval deposition (black arrows) per LPF (score 2) (H&E, x100); (**e**) Moderate inflammatory infilterate around the larva (score 2) (H&E, x400); (**f**) focally intact larval capsule (Masson trichrome, x400). (g-h) ROEO-treated (Group IV); (**g**) Muscle section showing mild inflammatory reaction around the larva (score 1) (H&E, x400); (**h**) Intact very thin larval capsule surrounding a degenerated larva (Masson trichrome, x400)

Examination of the diaphragmatic muscle sections of infected, non-treated mice (Group II) revealed a heavy larval density (score 3) harboring more than 10 larvae per LPF (Fig. 6a) and surrounded by intense inflammatory reaction (score 3) (Fig. 6b) as well as intact thick well developed larval capsule (Fig. 6c). On the contrary, the sections of infected ALB-treated mice (Group III) showed fewer larval deposition per LPF (score 2) (Fig. 6d) that were surrounded by a moderate inflammatory reaction (score 2) (Fig. 6e) with focally intact larval capsule (Fig. 6f). While those of infected ROEO-treated mice (Group IV) exhibited fewer larval deposition (score 2) surrounded by a mild inflammatory reaction (score 1) (Fig. 6g) and intact thin larval capsule (Fig. 6h).

### Biochemical study

Concerning serum MDA level in the intestinal phase, indeed *T. spiralis* infection resulted in a highly significant elevation of its levels with a mean of 17.5 ± 1.72 nmol/ml, however, treatment either with ALB or ROEO resulted in a remarkable statistically significant amelioration of the mean levels reaching 3.79 ± 0.66 nmol/ml and 4.25 ± 0.74 nmol/ml, respectively. A further elevation of MDA level in the muscular phase of infection was detected in the infected non-treated control group reaching a mean of 19.39 ± 1.58 nmol/ml. Again, both ALB and ROEO succeeded in reducing it, alas not reaching the normal levels, where they recorded 4.65 ± 0.76 nmol/ml and 6.14 ± 0.73 nmol/ml, respectively (Table 3).

**Table 3:** The effect of ALB and ROEO on the serum MDA level in nmol/ml in different studied groups compared to their controls

MDA (nmol/ml)\Group	Non infected Group	Infected Groups			F (*p*)
	Non-treated (Group I)	Non-treated (Group II)	ALB-treated (Group III)	ROEO-treated (Group IV)	
Mean ± SD	2.10 ± 0.53	**On 5th dpi**			289.080^*^ (< 0.001^*^)
		17.5 ± 1.72	3.79 ± 0.66	4.25 ± 0.74	
Median (Min - Max.)	2.13 (1.48–2.95)	17.3 (15–19.5)	3.83 (2.91–4.50)	4.45 (3.23–5.10)	
**p**_**0**_		< 0.001^*^	0.045^*^	0.008^*^	
**Significance**	p_1_ < 0.001^*^, p_2_ < 0.001^*^, p_3_ = 0.862				
Mean ± SD	2.10 ± 0.53	**On 28th dpi**			369.467^*^ (< 0.001^*^)
		19.39 ± 1.58	4.65 ± 0.76	6.14 ± 0.73	
Median (Min - Max)	2.13 (1.48–2.95)	19.4 (16.9–21.5)	4.77 (3.72–5.61)	6.17 (4.96–6.91)	
**p**_**0**_		< 0.001^*^	0.001^*^	< 0.001^*^	
**Significance**	p_1_ < 0.001^*^, p_2_ < 0.001^*^, p_3_ = 0.070				

F: for One way ANOVA test, Post Hoc Test (Tukey) for pairwise comparison

p: for comparing between the different studied groups

p_0_: for comparing non-infected non-treated (Group I) and each infected group

p_1_: for comparing II and III

p_2_: for comparing II and IV

p_3_: for comparing III and IV

*: Statistically significant at *p* ≤ 0.05

On the other hand, evaluation of the serum levels of GSH showed a significant reduction of its levels following the infection during the intestinal and muscular phases in both infected non-treated and infected ALB-treated mice. Nonetheless, a remarkable and highly statistically significant boosting of GSH levels following ROEO treatment in both intestinal and muscular phases was detected with a mean of 7.43 ± 0.84 mg/dl and 6.50 ± 0.75 mg/dl, respectively (*P* < 0.001) (Table 4).

**Table 4:** The effect of ALB and ROEO on the serum GSH level in mg/dl in different studied groups compared to their controls

Group\GSH (mg/dl)	Non infected Group	Infected Groups			F (*p*)
	Non-treated (Group I)	Non-treated (Group II)	ALB-treated (Group III)	ROEO-treated (Group IV)	
Mean ± SD	2.86 ± 0.15	**On 5th dpi**			218.254* (< 0.001*)
		1.39 ± 0.19	2.12 ± 0.22	7.43 ± 0.84	
Median (Min - Max.)	2.86 (2.65–3.10)	1.42 (1.15–1.61)	2.15 (1.77–2.37)	7.30 (6.57–8.51)	
**p**_**0**_		< 0.001^*^	0.044^*^	< 0.001^*^	
**Significance**	p_1_ < 0.049^*^, p_2_ < 0.001^*^, p_3_ < 0.001^*^				
Mean ± SD	2.86 ± 0.15	**On 28th dpi**			192.902* (< 0.001*)
		1.17 ± 0.14	1.85 ± 0.30	6.50 ± 0.75	
Median (Min - Max)	2.86 (2.65–3.10)	1.17 (0.95–1.37)	1.85 (1.33–2.16)	6.44 (5.54–7.38)	
**p**_**0**_		< 0.001^*^	0.002^*^	< 0.001^*^	
**Significance**	p_1_ < 0.049^*^, p_2_ < 0.001^*^, p_3_ < 0.001^*^				

F: for One way ANOVA test, Post Hoc Test (Tukey) for pairwise comparison

p: for comparing between the different studied groups

p_0_: for comparing non-infected non-treated (Group I) and each infected group

p_1_: for comparing II and III

p_2_: for comparing II and IV

p_3_: for comparing III and IV

*: Statistically significant at *p* ≤ 0.05

## Discussion

Trichinellosis was deemed a tantalizing human health and epizootiological problem [1]. The serious adverse reactions, pharmacokinetic and resistance flaws of front-line treatments have compromised their therapeutic efficacy [6]. The unresolved intricacies underscore the undeniable pressing need for natural safe therapeutic alternatives. From ancient times to the current era, medicinal essential oils have long loomed in the foreground of phytomedicine as proficient agents [39]. Owing to its astounding antioxidant potential, ROEO represents one of the most privileged phyto-essential oils [17].

Prior to the commencement of the in vivo study, an in-silico evaluation of the activity of the primary components of ROEO was performed. Molecular docking is a powerful computer tool for predicting and identifying probable binding modes between molecules and target proteins or receptors [40, 41]. The ROEO mechanisms of action against pathogens are complex as they are related to the presence of diverse molecules acting on discrete targets. For instance, two potential targets, tubulin tyrosine ligase [29] and thymidylate synthase [30], were identified to prove ROEO’s effectiveness against *T. spiralis* as a phytotherapeutic agent. Consistent with other studies, structural and functional disruptions of ROEO on cytoplasmic membrane, proton motive force, mitochondrial electron respiratory chain and DNA integration have been substantiated [42].

Parasitologically, the anti-*Trichinella* activity of ROEO was principally beholden on attributes of its chemical components. Our results concerning the anti-*Trichinella* activity of ROEO gave ground to such interpretation. ROEO compromised a plethora of compounds mainly monoterpenes, such as 1,8-cineole, pinene, camphor, camphene, and other oxygenated compounds as isoborneol that had successfully occupied the same binding pockets as ALB. In light of the previously described findings, the ROEO phytochemical components demonstrated promising nematocidal activity against both enzymes in line with the docking experiments. Phytochemicals of ROEO have been proved to suppress tubulin tyrosine ligase and thymidylate synthase leading to worm’s death. Our results were consistent with previously described studies that substantiated that inhibition of these enzymes induced cell apoptosis [29, 43–45]. The experimental results demonstrated that *T. spiralis* underwent cell apoptosis after being exposed to ROEO, which might confirm that the hypothesized mechanism of anthelmintic activity was due to the blockage of such enzymes. The lipophilic nature of monoterpenes aglycones allowed them to cross biological cellular membranes rapidly, interact with plenty of biomolecules and facilitate their entry [46, 47]. It is plausible to consider that the exhibited nematocidal activity of ROEO accounted for diverse mechanisms of actions extending from the ultrastructural to regulatory changes that have been postulated. Besides, the alteration in cellular membrane permeability, the present bioactive compounds contributed to the inhibited growth as they suppressed DNA compaction, disrupted the respiratory electrons chain thus reducing intracellular energy level and finally induced apoptosis [42]. This was corroborated by a study in which ROEO induced cellular and organelle membranes damage, mitochondrial depolarization, microtubule dysfunction, and arrested cell cycle at the G1/S phase, consequently triggering cell apoptosis [48]. In accordance with authors studying the in vitro antiparasitic activity of ROEO, promising antileishmanial as well as ovicidal and larvicidal potential on intestinal nematodes of sheep were reported [27, 42].

Parallelly, topographic disruptions induced by inhibitory binding of ALB to β-tubulin monomer in adult’s cuticle were following the corresponding reduction in worm burden [49]. However, modest changes were detected in ALB-treated muscular larvae as the fibrous encapsulation protected them and hindered their direct exposure to the drug [50]. On the other hand, marked ultrastructural changes induced by ROEO on both adult and larval stages of *T. spiralis* corresponded with the hypothesis that they were capable of altering the structure of the parasite. The deleterious changes are imputed most likely to the liposoluble nature of ROEO that enables its components to partite the lipids of cellular membranes and mitochondria rendering them more pliable and permeable. As a result, leakage of vital cell contents and ions caused electrolyte imbalance and eventually cell death [51, 52]. Even the non-dominant phytochemical components identified in ROEO such as camphene and isoborneol, could have been engaged in structural destruction as they inhibited thymidylate synthase, another potential *T. spiralis* target that catalyzes the reductive methylation of dUMP by N^5^,^10^-methylenetetrahydrofolate to produce thymidylate and dihydrofolate leading to cell apoptosis.

Indeed, *T. spiralis* adult worms induce an acute inflammatory immune response, goblet cells hyperplasia and villous atrophy in the intestinal mucosa [5]. However, non-resolution of intestinal damage induced by inflammation leads to development of associated pathology. It was plausible to utilize ROEO as a promising antiphlogistic phytotherapeutic agent to restrain *T. spiralis*-induced intestinal and muscular inflammation. The principle monoterpenes compounds were accountable for their anti-inflammatory attribute of ROEO [15]. Besides their high antioxidant activity, they suppress NF-κB transcription and the arachidonic acid pathway [53].

*T. spiralis* larval density in skeletal muscle commensurate with the severity of trichinellosis [8]. The larvae succeeded in transforming the infected myocytes into privileged nurse cells. For protection from the host immune response, the nurse cell is formed of host-derived cellular (mainly satellite cell) and collagenous components [54]. This fit well with that reported by another study which showed that oral administration of ALB to infected mice reduced larval count, inflammatory infiltrate and capsule thickness [55]. Building on the literature, disruption of nurse cell via antifibrotic agents is one of the successful mechanisms for interrupting the development of muscular larval stages. In the current study, mitigated inflammation and collagenous capsules formation in muscles were attributed to the antifibrotic attribute of ROEO. This thinning of the protective capsule could facilitate the entry of ROEO components into its interior and subsequently led to degeneration and death of the encapsulated larvae.

Apart from intestinal tissue, the skeletal muscle is specifically sensitive to any oxidant-mediated stress due to its high demand of oxygen [56]. The defensive host-derived antioxidants failed to overcome uncontrolled trichinellosis-mediated oxidative stress and detrimental muscular damage [57]. Excessive reactive oxygen species (ROS) produced by phagocytes impair both myogenesis and regeneration (satellite cells senescence and function) leading to progressive muscular atrophy by stimulating proteolysis and/or suppressing protein synthesis [56, 58]. Moreover, interference with redox homeostasis in striated muscles has a negative impact on membrane and DNA integrity as well as lipid and protein metabolism leading to the loss of function and death of myoblast [56]. As a consequence, MDA level in serum reflects any lipid peroxidation-mediated oxidative stress detected in the host during *T. spiralis* infection [56, 59]. In the current study, the marked increment in MDA and swift depletion of GSH sera levels in infected non-treated mice indicated that the released endogenous antioxidants failed to conquer the overwhelmed wave of radical-induced oxidation reaction. Our results were consistent with those who reported a rise in the serum MDA level in *T. spiralis*-infected in the first four weeks [60]. Compliant with another study that reported that the intensification of serum antioxidant activity was observed during the third to seventh week pi corresponding to the maximal muscle damage induced by *T. spiralis* larvae, that stimulated phagocytes to produce ROS [61]. In our study, the slight increment in GSH level after ALB treatment is ascribed to the consumption of endogenous released GSH to curtail slight damage induced by low worm burden. These results fit well with others who attributed the slight rise in serum GSH to the marked reduction in worm burden and subsequently minimal damage induced in infected ALB-treated mice [62]. Following oral administration of ROEO, the MDA level was lowered accompanied by higher increase in serum GSH level. The increment in GSH serum level could be ascribed to its eminent high antioxidant activity of ROEO. These data provide strong evidence that the appealing high antioxidant activity of ROEO was capable of ameliorating oxidant-mediated damage by neutralization of inflammatory ROS [63]. ROEO was rich in monoterpenes accounting for the antioxidant activity shown [64].

## Conclusions

Based on the presented data, we conclude that ROEO was a proficient phytotherapeutic agent via mediating potent multistage anti-*Trichinella* and would show antioxidant, anti-inflammatory, and antifibrotic activities. This study is the first one to highlight the potential therapeutic efficacy of ROEO in experimental murine trichinellosis via in silico and in vivo studies. Building on our results, further studies are required to explore the promising trichinocidal activity of the individual phytochemicals of ROEO as well as comparing the different chemotypes.

## Data Availability

Data is provided within the manuscript.
